# Whole-genome resequencing reveals genetic diversity and selection signals in warm temperate and subtropical *Sillago sinica* populations

**DOI:** 10.1186/s12864-023-09652-3

**Published:** 2023-09-15

**Authors:** Xiang Zhao, Tianlun Zheng, Tianxiang Gao, Na Song

**Affiliations:** 1 The Key Laboratory of Mariculture (Ocean University of China), Ministry of Education, Qingdao, 266003 Shandong China; 2 Zhejiang Fisheries Technical Extension Center, Hangzhou, 310023 Zhejiang China; 3 Fishery College, Zhejiang Ocean University, Zhoushan, 316022 Zhejiang China

**Keywords:** *Sillago sinica*, Genetic diversity, Selective sweep, Environmental adaptability

## Abstract

**Background:**

Genetic diversity and heterogeneous genomic signatures in marine fish populations may result from selection pressures driven by the strong effects of environmental change. Nearshore fishes are often exposed to complex environments and human activities, especially those with small ranges. However, studies on genetic diversity and population selection signals in these species have mostly been based on a relatively small number of genetic markers. As a newly recorded species of Sillaginidae, the population genetics and genomic selection signals of *Sillago sinica* are fragmented or even absent.

**Results:**

To address this theoretical gap, we performed whole-genome resequencing of 43 *S. sinica* individuals from Dongying (DY), Qingdao (QD) and Wenzhou (WZ) populations and obtained 4,878,771 high-quality SNPs. Population genetic analysis showed that the genetic diversity of *S. sinica* populations was low, but the genetic diversity of the WZ population was higher than that of the other two populations. Interestingly, the three populations were not strictly clustered within the group defined by their sampling location but showed an obvious geographic structure signal from the warm temperate to the subtropics. With further analysis, warm-temperate populations exhibited strong selection signals in genomic regions related to nervous system development, sensory function and immune function. However, subtropical populations showed more selective signalling for environmental tolerance and stress signal transduction.

**Conclusions:**

Genome-wide SNPs provide high-quality data to support genetic studies and localization of selection signals in *S. sinica* populations. The reduction in genetic diversity may be related to the bottleneck effect. Considering that low genetic diversity leads to reduced environmental adaptability, conservation efforts and genetic diversity monitoring of this species should be increased in the future. Differences in genomic selection signals between warm temperate and subtropical populations may be related to human activities and changes in environmental complexity. This study deepened the understanding of population genetics and genomic selection signatures in nearshore fishes and provided a theoretical basis for exploring the potential mechanisms of genomic variation in marine fishes driven by environmental selection pressures.

## Background

A central challenge in evolutionary ecology is to identify the underlying genomic variation in natural populations responsible for environmental adaptation [1, 2]. Quite a few studies have focused on several organisms such as plants, crops, and terrestrial animals [3–5], but studies on genomic variation in native adaptations of marine fishes are somewhat fragmented. Compared to terrestrial environments, marine environments may be more complex and variable in local areas [6]. Therefore, under the influence of global climate change and human activities, the evolution of the marine environment may be more rapid and unpredictable. It is obvious that, in view of this, marine fishes, the ancient ectothermic vertebrates, will be under great pressure to adapt to the environment. Although biological populations respond by adjusting their distribution and reproduction patterns [7], a significant proportion of populations do not respond sufficiently to counteract environmental changes, a process considered to be the first step towards species extinction [8]. However, to some extent, this environmental stress may also be an important evolutionary driver for some fish. Evolutionary drive or extinction depends on whether fish accumulate sufficient genetic variation in beneficial traits since the generation of sustained variation or new mutations is decisive for organisms to adapt to rapidly changing environments [9]. More recently, there has been growing interest in exploring potential genomic variation in natural populations associated with environmental change and changes in the genetic diversity of populations in different environments. To date, although the links between genomic variation and local environmental adaptation have been reasonably well studied in the field of landscape genetics of terrestrial taxa [10, 11], far less is known about these relationships in marine ecosystems, especially for some newly identified species of marine fish.

Marine fish often inhabit a wide variety of complex and changing environments [12]. However, for aquatic ectotherms, ambient temperature is predicted to be an important driver of local adaptation due to the strong dependence of body temperature on water temperature [13]. There are still many difficulties and uncertainties in identifying genomic variants associated with local environmental adaptation. In recent years, the rapid development of whole-genome resequencing technologies and the continuous improvement of gene function databases have enabled researchers to rapidly resolve genome-wide variation loci in populations [14]. In addition, quantitative genetics approaches support the use of species-specific trait data as a linkage to associate genomic variation associated with environmental adaptations. For example, whole-genome resequencing-based single nucleotide polymorphisms (SNPs) in inland and marine *Coilia nasus* populations revealed differences in immune, visual, migratory and osmoregulation between these two ecotypes [15]. Whole-genome resequencing and a phenotype-specific genome-wide association study (GWAS) revealed the possible involvement of ceramide kinase genes related to cardiac function in thermal adaptation in wild redband trout (*Oncorhynchus mykiss gairdneri*) populations [16]. The large number of variant loci at the genome-wide level provides a wealth of available data to reveal the genetic diversity and local environmental adaptations of biological populations. The large number of samples and experimental basis required for specific adaptive phenotypes may pose a degree of limitation for studies in some fish in which samples are more difficult to obtain. However, considering the combination of complex aquatic environmental factors, it is still possible to predict adaptation processes in different ecotypes by gene function in regions of genomic variation among different populations, although this is sometimes limited by molecular functional databases.

*S. sinica*, is a newly identified species belonging to Sillaginidae (Perciformes), that was found in the inshore waters from the Bohai Sea to northern part of the South China Sea. [17]. This species is euryhaline but does not migrate over long distances and adult fish will bury themselves in the sand when disturbed [18]. By exploring the sequence of the mitochondrial control region of *S. sinica*, Xue found that there was no random mating between its different populations, which might be related to the living habits of *S. sinica* [19]. Similar results were found from population genetics studies based on microsatellite sequences [20]. These results suggest that *S. sinica* might be an ideal model for exploring local adaptation. Key loci for local environmental adaptations in *Sillago japonica*, a close relative of *S. sinica*, have been explored by whole-genome resequencing and species distribution models, and the results revealed that candidate genes related to membrane fluidity may underlie their adaptation to cold environments [21]. As a newly recorded species of Sillaginidae, the genomic loci of local environmental adaptations in the *S. sinica* populations remain largely unexplored. Our laboratory has assembled the complete genome map of *S. sinica* in the previous stage [22], which has laid the data basis for exploring its population genetic diversity and genome-wide variant loci.

In the present study, we sequenced the whole genomes of *S. sinica* individuals collected from three sites across the coastal waters of China, covering warm temperate (Yellow Sea, Qingdao, QD; Bohai Sea, Dongying, DY) and subtropical (East China Sea, Wenzhou, WZ) populations. The aims of this study were, to summarize, (1) to reveal differences between populations’ genetic diversity and genetic structure based on the obtained genome-wide variation data. (2) to clarify effective population size and evolutionary history, and (3) to infer local adaptive mechanisms based on selection signals and gene function. To our knowledge, this may be the first study to sequence the whole genome of *S. sinica* and may provide a new perspective on its population genetics and local environmental adaptation.

## Results

### Sequencing information and variation calling.

**Fig. 1:**
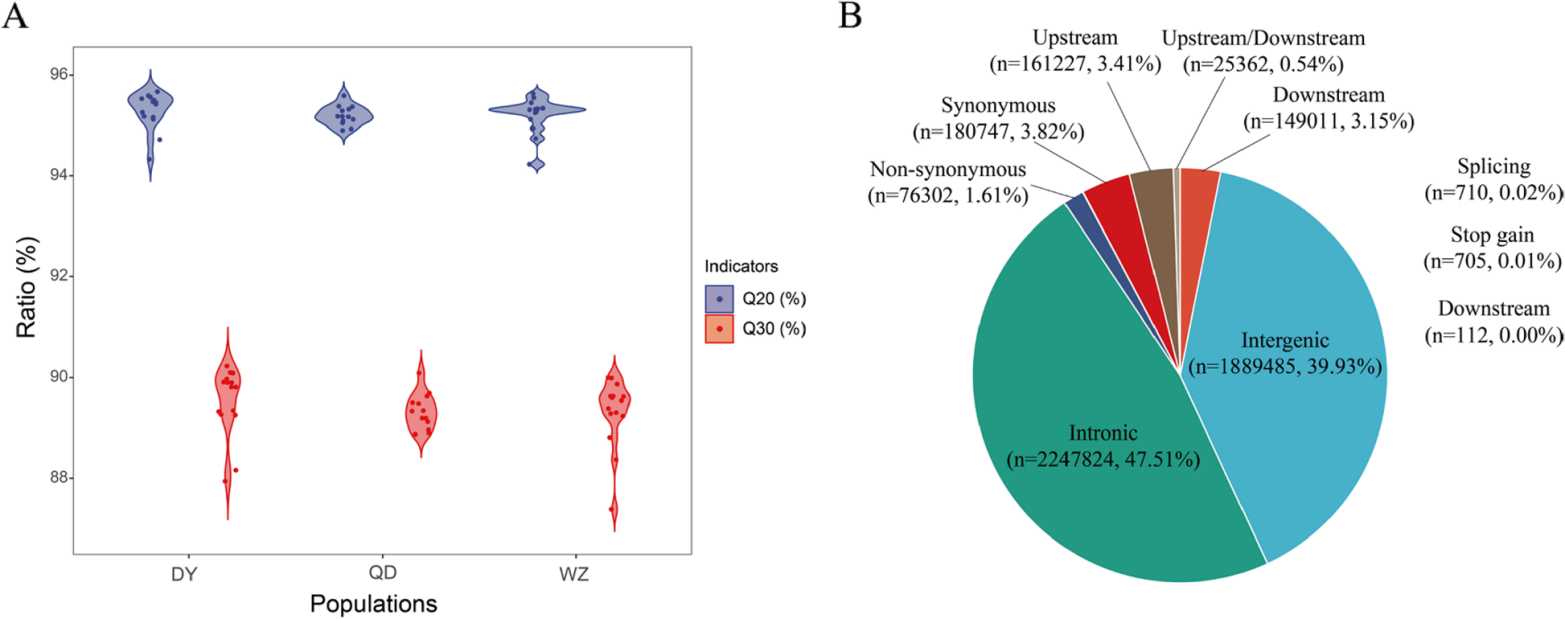
Q20 and Q30 of the clean reads (**A**). Statistical results of SNP detection (**B**)

Deep resequencing of the 43 samples of *S. sinica* generated a total of 576.43 G raw data, with an average of 13405.42 M raw data per sample, and a total of 574.60 G of filtered clean data, with an average of 13362.97 M per sample. The Q20 and Q30 of the clean reads were both approximately 90% (Fig. 1A). The average depth was 20.65 × (18.04‒28.16 ×) for 15 DY individuals, 21.28 × (16.21‒30.73 ×) for 13 QD individuals, and 22.03 × (17.29‒32.93 ×) for 15 WZ individuals. In addition, 98.53% of the sequencing reads were mapped to the *S. sinica* reference genome. We obtained a total of 11,007,756 raw SNPs after mapping. After filtering and screening, a total of 4,878,771 high-quality SNPs were obtained. Most of the SNPs were located in the intronic and intergenic regions (Fig. 1B).

### Population structure, linkage disequilibrium, and historical dynamics

**Fig. 2:**
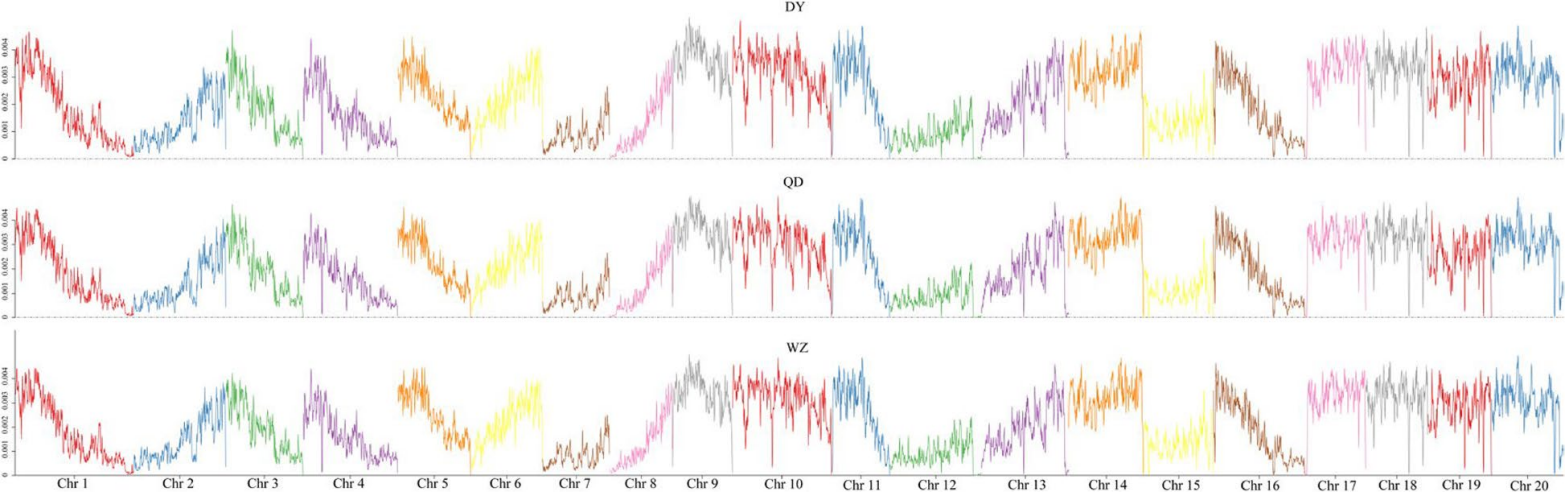
Genome-wide sliding window analysis of population nucleotide diversity

**Fig. 3:**
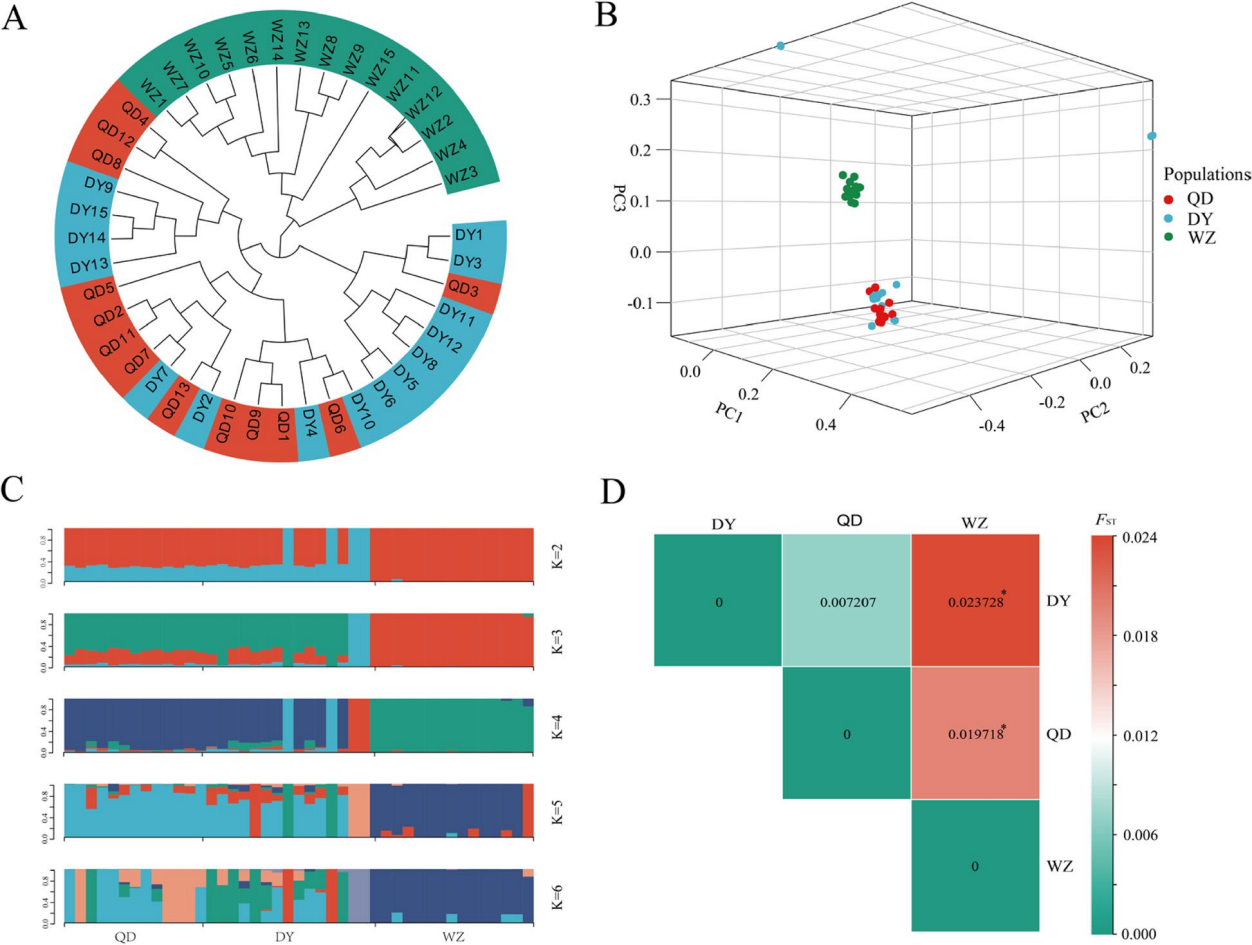
Neighbor-joining tree constructed using *p*-distances of 43 *S. sinica* individuals (**A**). Principal component analysis for 43 *S. sinica* individuals (**B**). Admixture analysis of three *S. sinica* populations (**C**). The length of each coloured segment represents the proportion of individual genomes inferred from ancestral populations (K = 2–6). *F*st values between three *S. sinica* populations. “*” represents *P* < 0.05 (**D**)

**Fig. 4:**
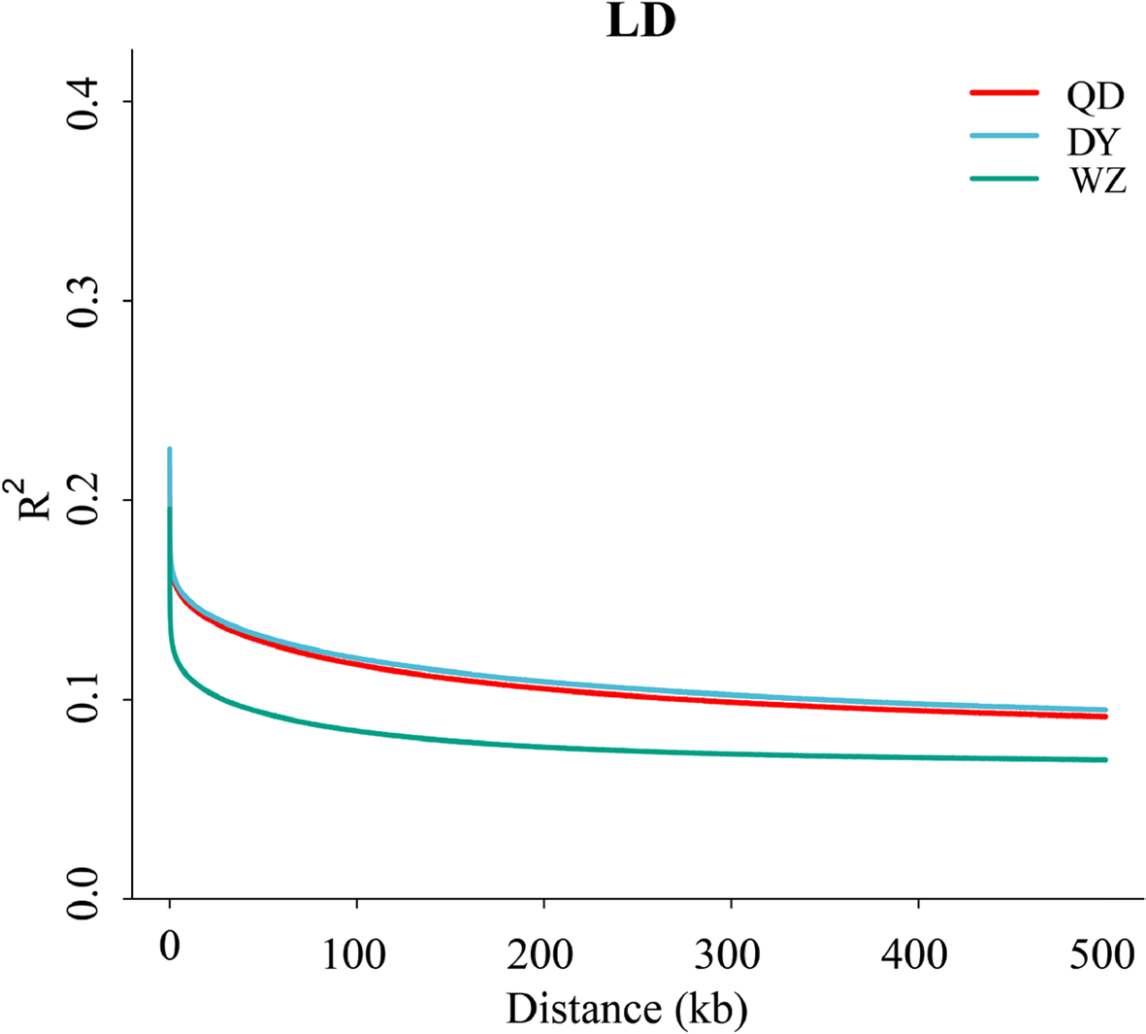
Decay of linkage disequilibrium in the QD, DY and WZ populations

**Fig. 5:**
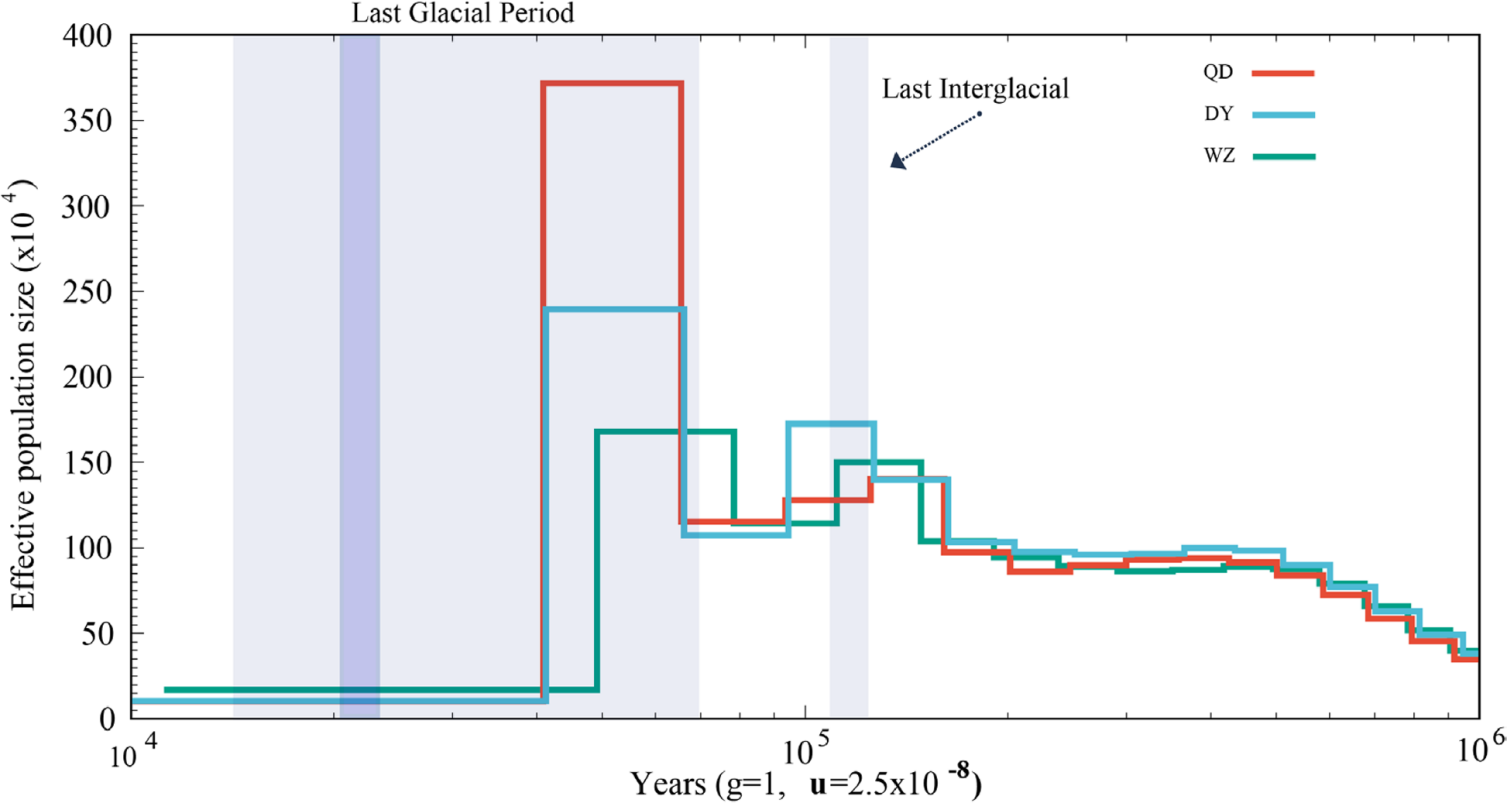
Demographic history of three *S. sinica* populations in this study

**Fig. 6:**
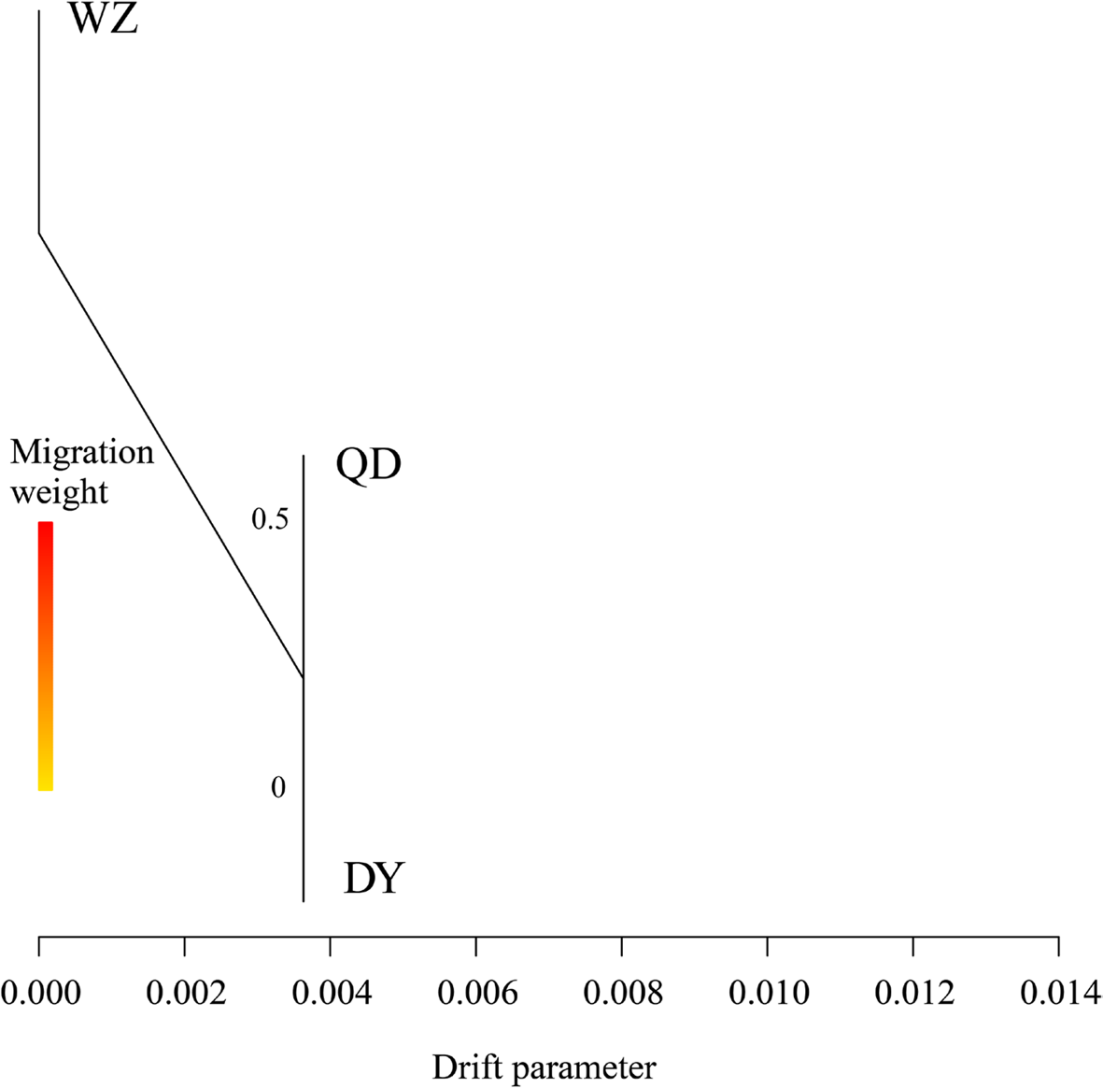
Maximum likelihood relationships among 3 populations calculated with Treemix (no gene migration)

The mean nucleotide diversity (π) of the WZ, QD and DY populations was calculated to be 0.002444, 0.002417, and 0.002418, respectively (Fig. 2). A neighbor-joining (NJ) tree was constructed based on the whole-genome SNPs. The NJ tree showed that all the individuals were clustered into two large clades, the QD and DY populations were clustered into one clade and the WZ populations were clustered into the other clade (Fig. 3A). The results of the principal component analysis (PCA) presented similar clusters as the NJ tree. The first principal component (PC1) separated the subtropical (WZ) and warm temperate clades (QD and DY), consistent with the NJ tree result. None of the first, second and third principal components (PC1, PC2 and PC3) separated the QD and DY populations, which is consistent with the mixed clustering results in the NJ tree (Fig. 3B). Admixture analysis further supported the phylogenetic tree and PCA results (Fig. 3C). At K = 2, K = 3 and K = 4, the WZ population both formed a separate cluster. The clusters were shared by both QD and DY populations in the results for all K values. At K = 5 and K = 6, the WZ population was separated as a unique cluster, and the individuals of the QD and DY populations were highly mixed. We calculated the *F*_ST_ between populations based on all SNPs and then averaged them. Both the QD and QY populations showed significant genetic divergence from the WZ population (Fig. 3D). In total, three populations were not strictly clustered within the group defined by their sampling location, but showed an obvious geographic structure signal from the warm temperate to subtropics. Linkage disequilibrium (LD, measured as R^2^) decreased to half of its maximum value at 31.2 kb in the WZ population but at 51.2 kb and 57.5 kb in the QD and DY populations, respectively (Fig. 4). Pairwise sequentially markovian coalescent (PSMC) analysis revealed that the three *S. sinica* populations have experienced two bottleneck effects during the last one million years, occurring in the last interglacial and pre-last glacial periods. The effective population size (*Ne*) of the WZ population reached a minimum of 5 × 10^4^ years ago, while the minimum *Ne* values of QD and DY populations were 4 × 10^4^ years ago (Fig. 5). The ML tree without migration events inferred from TreeMix analysis divided the 43 individuals into two clusters, similar to the population structuring patterns identified from the NJ phylogenetic tree, PCA, and genetic structure analysis (Fig. 6).

### Genome-wide selection signal and candidate gene analysis based on *F*st&π

**Fig. 7:**
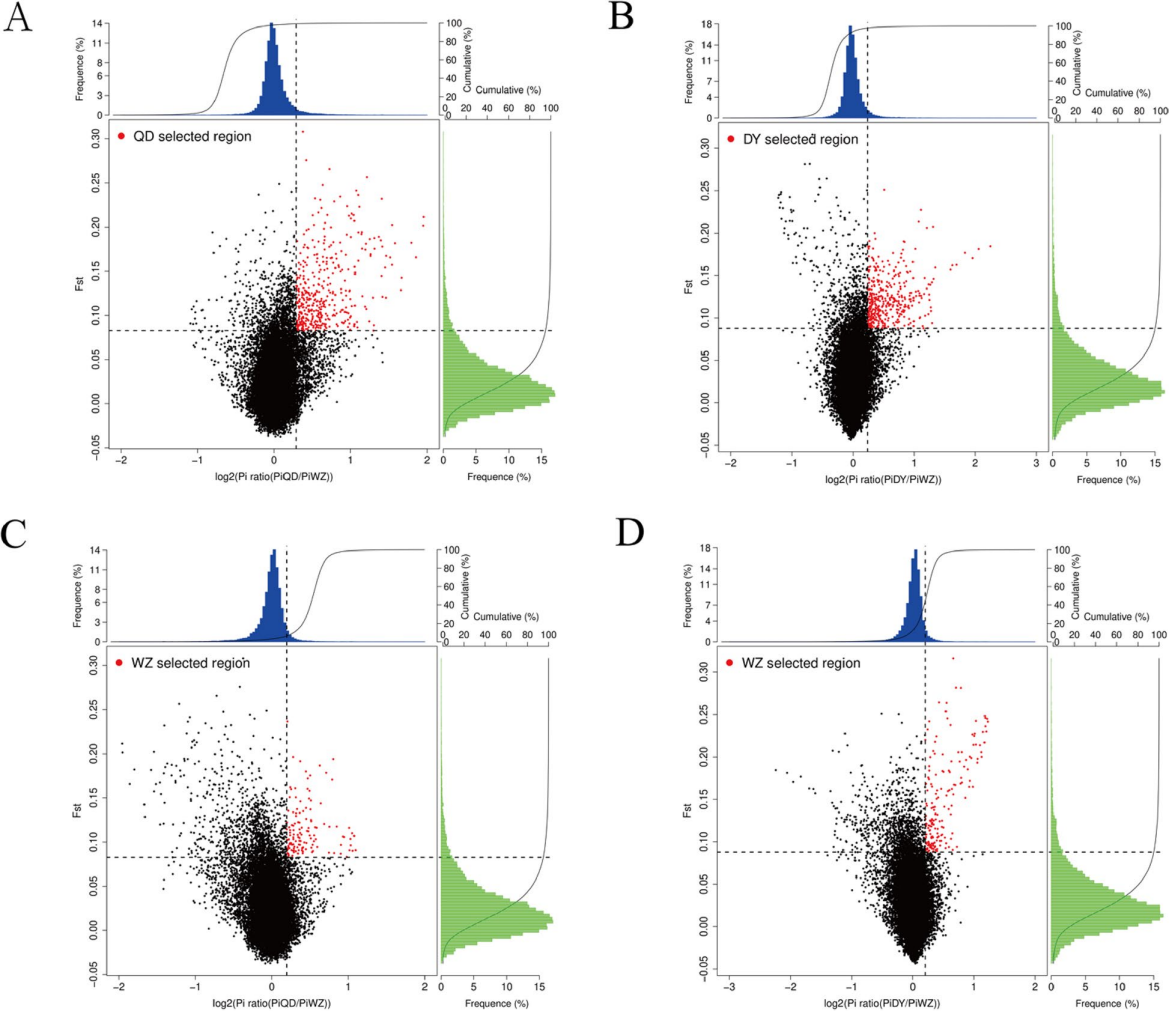
Genomic regions with strong selective signals in populations of *S. sinica*. Note: Distribution of log_2_(π ratios) and *F*st values calculated in 40 kb sliding windows with 20 kb increments between QD/WZ populations (WZ as the control group) (**A**). Distribution of log_2_(π ratios) and *F*st values between DY/WZ populations (WZ as the control group) (**B**). Distribution of log_2_(π ratios) and *F*st values between WZ/QD populations (QD as the control group) (**C**). Distribution of log_2_(π ratios) and *F*st values between QD/WZ populations (WZ as the control group) (**D**)

**Fig. 8:**
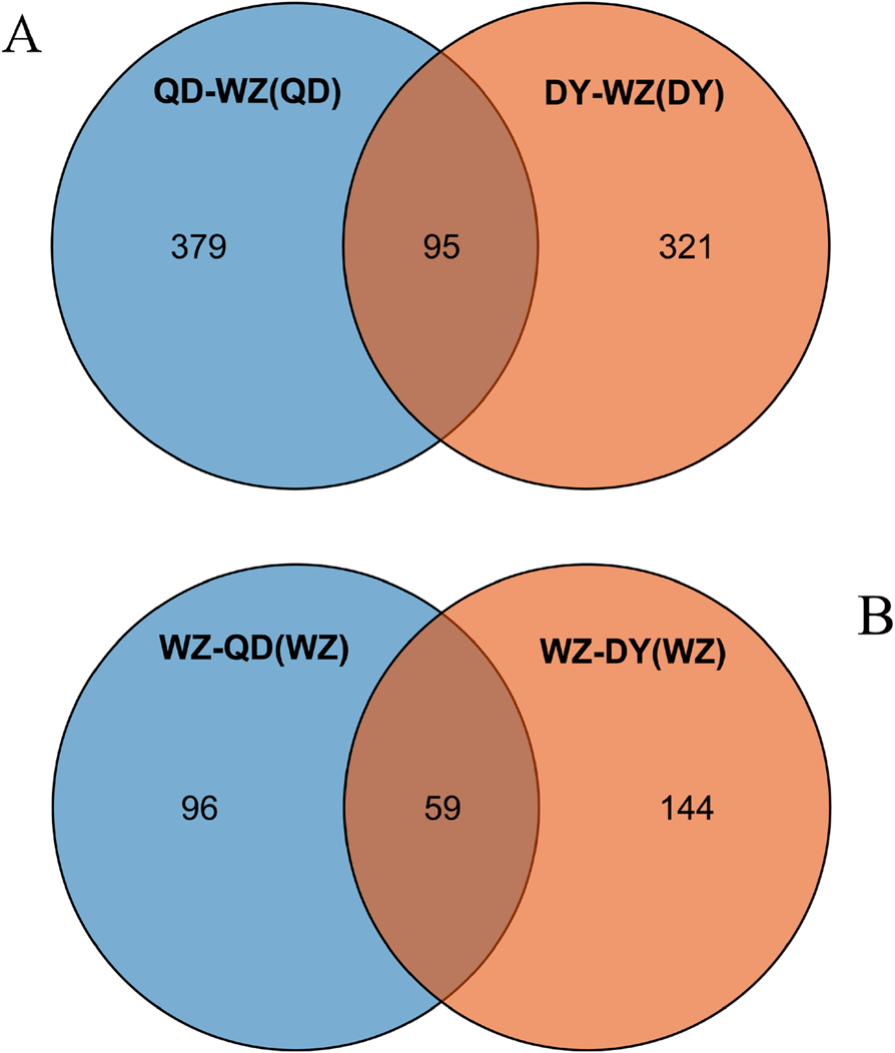
Overlapping warm temperate adaptation genes in QD/WZ and DY/WZ pairs based on the Venn diagram (**A**). Overlapping subtropical adaptation genes in WZ/QD and WZ/DY pairs (**B**)

We detected strong selection signals in the genome based on *F*st&π to mine functional regions that are closely related to the survival environment. Considering that there were no significant genetic differences between the QD and DY populations, we selected the WZ population as a control group to reveal candidate warm temperate environmental selection genes in the QD and DY populations. Using the top 5% of maximum *F*st (*F*st ≥ 0.0834) and π ratio (π_QD/WZ_ ≥ 0.2813) values, a total of 474 candidate genes (corresponding to 7.78 Mb in size) were identified in the QD population (Fig. 7A). Similarly, 416 candidate genes (corresponding to 6.68 Mb in size) (*F*st ≥ 0.0891 and π_DY/WZ_ ≥ 0.2821) were identified in the DY population (Fig. 7B). We identified 95 overlapping genes between the QD and DY populations as potential genes associated with adaptation to warm temperate environments (Fig. 8A). Compared with the QD and DY populations, we obtained 155 and 203 candidate genes in the WZ population, respectively (Fig. 7C and D). A total of 59 overlapping genes were identified as potential genes associated with adaptation to subtropical environments (Fig. 8B).

**Fig. 9:**
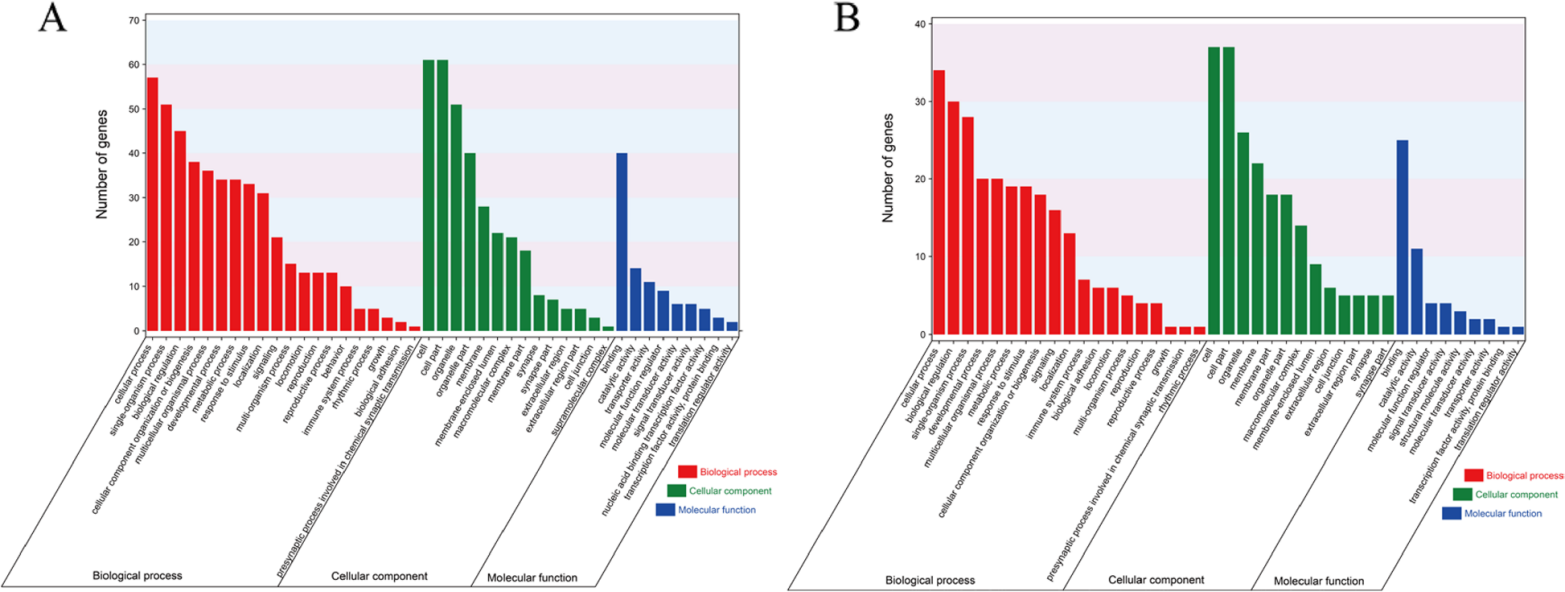
GO classification for warm temperate adaptation genes (**A**). GO classification for subtropical adaptation genes (**B**)

**Fig. 10:**
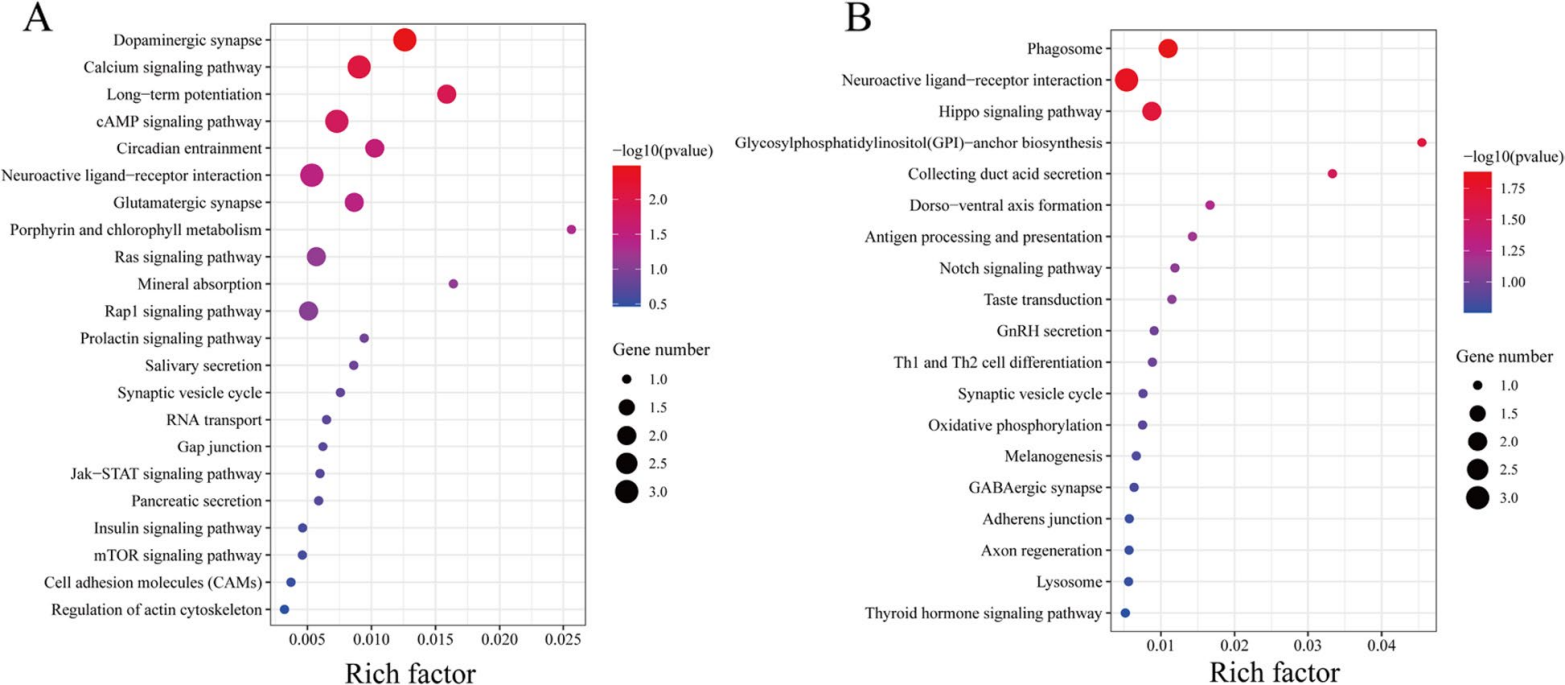
KEGG enrichment for warm temperate adaption genes (**A**). KEGG enrichment for subtropical adaption genes (**B**)

To obtain a broad overview of the molecular functions of these potential genes associated with adaptation to environments, we performed Gene Ontology (GO) and Kyoto Encyclopedia of Genes and Genomes (KEGG) enrichment analyses on the candidate genes. GO analysis showed that the main GO terms for the potential genes associated with adaptation to warm temperate and subtropical environments included cellular process (GO: 0009987) and single-organism process (GO: 0044699) in biological processes, cell (GO: 0005623) and cell part (GO: 0043226) in cellular component, binding (GO: 0005488) and catalytic activity (GO: 0003824) in molecular function (Fig. 9). KEGG enrichment analysis showed that 95 warm temperate adaptation candidate genes were significantly enriched in the dopaminergic synapse (ko04728, *p* = 0.00), calcium signaling pathway (ko04020, *p* = 0.00), long-term potentiation (ko04720, *p* = 0.01), cAMP signaling pathway (ko04024, *p* = 0.01), circadian entrainment (ko04713, *p* = 0.03), neuroactive ligand-receptor interaction (ko04080, *p* = 0.03) and glutamatergic synapse (ko04724, *p* = 0.03). However, 59 subtropical adaption candidate genes were significantly enriched in the phagosome (ko04145, *p* = 0.01), neuroactive ligand-receptor interaction (ko04080, *p* = 0.01), Hippo signaling pathway (ko04390, *p* = 0.02), glycosylphosphatidylinositol (GPI)-anchor biosynthesis (ko00563, *p* = 0.02), and collecting duct acid secretion (ko04966, *p* = 0.03) (Fig. 10).

### Genome-wide selection signal and candidate gene analysis based on XP-EHH

**Fig. 11:**
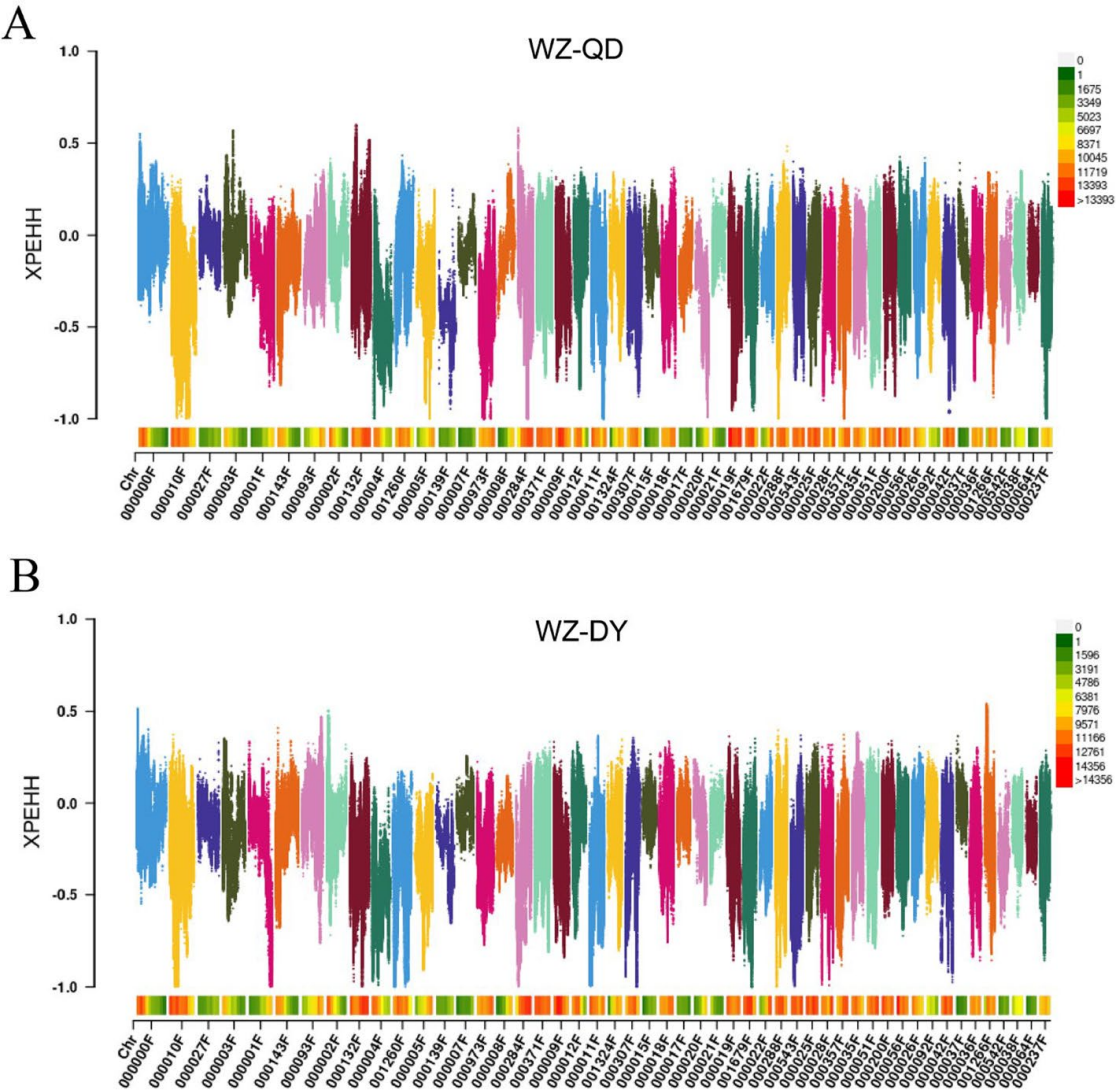
XP-EHH analysis between the WZ and QD populations (**A**). XP-EHH analysis between the WZ and DY populations (**B**)

**Fig. 12:**
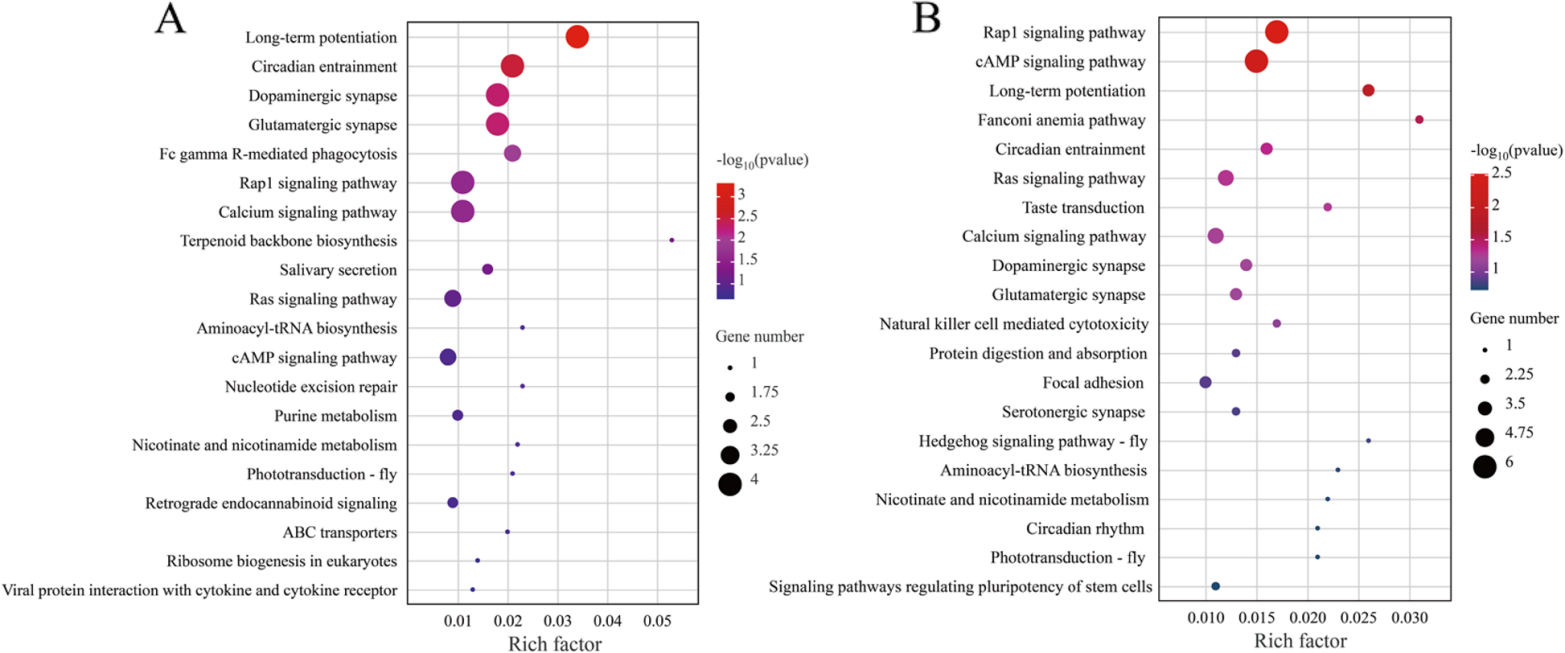
KEGG enrichment for the selected genes based on XP-EHH values between the WZ and QD populations (**A**). KEGG enrichment for the selected genes based on XP-EHH values between the WZ and DY populations (**B**)

Based on the theory of linkage disequilibrium, the degree of linkage disequilibrium between loci decreases gradually with increasing distance between markers. Therefore, different lengths of extended haplotype homozygousity (EHH) caused by selection can be observed on the genome. We used the XP-EHH (cross population extended haplotype homozogysity) method to detect selection signals between populations. No strong selection signals were detected between QD and DY populations. We set "XP-EHH < -1" between the WZ and QD populations to screen for selected loci and obtained 83 genes (Fig. 11A). Similarly, 104 genes were obtained based on the XP-EHH values between WZ and DY populations (Fig. 11B). KEGG enrichment analysis showed that selected genes between WZ and QD populations were significantly enriched in long-term potentiation (ko04720, *p* = 0.00), circadian entrainment (ko04713, *p* = 0.00), dopaminergic synapse (ko04728, *p* = 0.00), glutamatergic synapse (ko04724, *p* = 0.00), FC gamma R-mediated phagocytosis (ko04666, *p* = 0.01), Rap1 signaling pathway (ko04015, *p* = 0.02) and calcium signaling pathway (ko04020, *p* = 0.02) (Fig. 12A). The selected genes between WZ and DY populations were significantly enriched in the Rap1 signaling pathway (ko04015, *p* = 0.00), cAMP signaling pathway (ko04024, *p* = 0.01), long-term potentiation (ko04720, *p* = 0.01), Fanconi anemia pathway (ko03460, *p* = 0.03), circadian entrainment (ko04713, *p* = 0.04), and Ras signaling pathway (ko04014, *p* = 0.04) (Fig. 12B). Interestingly, these results were generally consistent with the *F*st&π analysis.

## Discussion

In this study, we compared three populations (DY, QD and WZ from coastal China) based on genetic diversity, population structure, and selective signals, with a focus on genomic variations. Previous studies have identified a few traditional molecular markers for *S. sinica*, such as mitochondrial DNA and microsatellite markers [18]. However, genomic research on *S. sinica* populations has been limited largely by the lack of abundant applicable molecular markers compared to other fish. The labor-wasting, limited numbers and genetic information of traditional markers make population genetic research difficult [23]. SNP markers, as emerging molecular markers, provide an essential alternative method to study genetic diversity, genetic structure and selection signals in biological populations [24]. Many population genetic analyses of various fish, such as *Oreochromis niloticus*, *Micropterus salmoides* and *Larimichthys polyactis* have been carried out using SNP markers [25–27]. This study is the first large-scale SNP development effort in *S. sinica* to our knowledge. It has been shown that a sufficient number of SNPs can be obtained from second-generation high-throughput sequencing data at 15 × sequencing depth [28]. In this study, the average sequencing depths of individuals in the DY, QD, and WZ populations were 20.65 × , 21.28 × and 22.03 × , respectively, and the lowest sequencing depth was for a single individual (16.21 ×). In addition, the mapping rate of all samples to the reference genome was approximately 98%. The above indicators prove that the SNP data obtained in this study are sufficient and reliable.

### Population genetic analysis and historical dynamics

According to the theory of genetics and evolutionary biology, genetic diversity is the basis and core of biodiversity and the fundamental guarantee of the evolutionary potential of species [29]. Several studies have shown that the higher the genetic diversity, the more adaptable it is to the environment. A decrease in genetic diversity can lead to a decrease in the adaptability of a species to its environment [30]. Several recent studies have investigated the nucleotide diversity of *S. sinica* populations based on mitochondrial DNA, but the results are not always consistent. For example, Zhang et al. found that the nucleotide diversities of the WZ, QD and DY *S. sinica* populations were 0.012, 0.005 and 0.001, respectively [31]. However, a similar study found nucleotide diversity in the WZ, QD and DY populations to be 0.016, 0.007 and 0.014, respectively [32]. Although the values are not the same, these studies convey an important signal that the genetic diversity of *S. sinica* populations is at a low level, but the genetic diversity of the WZ population is higher than that of the other two populations. Our results validated previous main findings and extended these earlier results to genome-wide variant loci. Linkage disequilibrium analysis seems to support the genetic diversity results. Studies have shown that if the population undergoes positive selection, the frequency of the surrounding sites linked to the favorable site will increase rapidly due to the piggyback effect, so the haplotype containing the favorable site has a higher frequency on the one hand, and on the other hand, because the duration is short, it also has a long range of LD influence [33]. This feature provides an effective breakout point for detecting whether positive selection has occurred. Our results showed that both the DY and QD populations had higher LDs than the WZ populations, suggesting that the DY and QD populations were subject to higher selection intensity and lower genetic diversity [34]. This phenomenon of genetic diversity appears to be explained by population history dynamic outcomes. Our results suggested that three *S. sinica* populations experienced the first bottleneck effect during the last interglacial (1.1–1.3 × 10^5^ years ago; a relatively warm geological period), with a sharp decline in effective population size. Although the population size of the *S. sinica* rapidly increased during the pre-Last Glacial Period, the genetic diversity of the *S. sinica* populations did not increase sufficiently, as there are highly similar “redundant copies” in the increased number of individuals from a genetic point of view [35]. This explanation is reasonable and intriguing, but further direct evidence is needed.

Considering the existence of geographical isolation, the grouping of species is usually based on the geographical location of sampling first, but the flaw of this method usually breaks our logical chain. The results of population genetic structure research based on genome-wide variation loci are usually incompatible with geographical grouping. In this study, the NJ tree showed that all the individuals were clustered into two large clades: the QD and DY populations were clustered into one clade and the WZ populations were clustered into the other clade. The PCA and admixture results also showed confounding effects of the QD and DY populations. The population genetic differentiation index can serve as further evidence. Our results showed no significant genetic differentiation between the QD and DY populations, but both populations developed a moderate degree of genetic differentiation from the WZ population. This result is inconsistent with the results of mitochondrial DNA [32]. The most reasonable explanation is that the mitochondrial DNA sequence is too short and contains limited genetic information. In addition, no gene migration events were detected between the 3 populations, but the ML tree without migration events inferred from TreeMix analysis divided the 43 individuals into two clusters. In total, three populations did not strictly cluster within the group defined by their sampling location, but showed an obvious geographic structure signal from the warm temperate to subtropics.

### Population selection signals based on *F*st&π

We detected strong selection signals in the genome based on *F*st&π to identify functional regions that are closely related to the survival environment. Considering the significant genetic structure of the three *S. sinica* populations, we defined the QD and DY populations as warm temperate populations to screen for selection signals. We identified 95 overlapping genes between the QD and DY populations as potential genes associated with adaptation to warm temperate environments. KEGG enrichment results showed that these genes were related to environmental adaptation, the nervous system, signal transduction and signaling molecules and interactions. Dopaminergic synapse, long-term potentiation and glutamatergic synapse pathways are considered to be related to the development of the nervous system, vision and hearing of fish [36–38]. We identified two key genes involved in these processes, namely GRIN2A (glutamate receptor) and DRD5 (D1B dopamine receptor). Glutamate receptor have been shown to play an important role in excitatory synaptic transmission in the biological central nervous system as ligand-gated ion channels [39]. The D1B dopamine receptor plays a key receptor role in biological brain and neural development [40]. Although these two genes have not been confirmed in fish studies, the results of the present study imply that they may be evidence of the adaptability of the nervous system and sensory function of the *S. sinica* warm temperate populations. Selection signals in the nervous system may be related to reduced environmental complexity due to human activities, but the deeper mechanisms need to be further explored [41].

We defined the WZ population as the subtropical population to screen for selection signals. A total of 59 overlapping genes were identified as potential genes associated with adaptation to subtropical environments. KEGG enrichment results showed that these genes were related to transport and catabolism, signaling molecules and interactions, signal transduction, glycan biosynthesis and metabolism and excretory system. We identified a key gene in the phagosome pathway, ATP6V1B2 (V-type proton ATPase subunit B), which was shown to help fish acidify external media to improve environmental ammonia tolerance [42]. This selection signal may suggest a role for human activities and coastal engineering, but the specific environmental elements need further investigation. In addition, we identified three genes related to stress signaling in neuroactive ligand-receptor interaction and the Hippo signaling pathway, namely HRH4 (histamine H4 receptor), GABBR1 (gamma-aminobutyric acid type B receptor) and GALR1 (galanin receptor type 1). The living environment of nearshore demersal fish is more complex, with frequent changes in temperature, salinity, turbidity and other environmental factors. The long-term changes in these environments may cause the adaptive evolution of fish stress responses. Elicitation of these stress responses requires signal perception, signal transduction and large-scale gene expression changes [43]. The selection signal of the WZ population may suggest the complexity of its survival environment, and combined with the results of genetic diversity, we hypothesized that this environmental complexity may be the evolutionary driver of local adaptation.

### Population selection signals based on XP-EHH

 We used the XP-EHH method to further detect strong selection signals between populations. No strong selection signals were detected between the QD and DY populations. We detected 83 genes between the WZ and QD populations, which were mainly associated with the nervous system, environmental adaptation, immune system, and signal transduction. Based on the KEGG enrichment results, we again localized to the GRIN2A gene. In addition, guanine nucleotide-binding protein G (q) (GNAQ) and dedicator of cytokinesis protein 2 (DOCK2) were also identified as candidate genes. GNAQ is involved as a modulator or transducer in various transmembrane signaling systems [44]. DOCK2 has been shown to be involved in the migration and motility of lymphocytes, suggesting that warm temperate and subtropical populations have strong selection signals on genes related to immune regulation [45]. A total of 104 genes were obtained between the WZ and DY populations which were mainly associated with signal transduction, the nervous system, DNA replication and repair, and environmental adaptation. Interestingly, we again localized to the GRIN2A gene. In addition, 5-hydroxytryptamine receptor 1B (HTR1B), the G-protein coupled receptor for 5-hydroxytryptamine, can regulate the release of 5-hydroxytryptamine, dopamine and acetylcholine in the vertebrate brain, and thereby affect neural activity, mood and behaviour [46]. Although the HTR1B gene has been less studied in fish, the results of the present study imply that it may be evidence of differences in the nervous system and sensory function between the *S. sinica* warm temperate and subtropical populations. In conclusion, these results further support the results of the *F*st&π analysis.

## Conclusions

Genome-wide SNPs provide high-quality data to support genetic studies and the localization of selection signals in *S. sinica* populations. In the present study, we detected low levels of genetic diversity in the QD, DY and WZ populations. The reduction in genetic diversity may be related to the bottleneck effect. Considering that low genetic diversity leads to reduced environmental adaptability, conservation efforts and genetic diversity monitoring of this species should be increased in the future. Interestingly, the three populations did not strictly cluster within the group defined by their sampling location, but showed an obvious geographic structure signal from the warm temperate to subtropics. Warm-temperate populations exhibit strong selection signals in genomic regions related to nervous system development, immune regulation and sensory function. However, subtropical populations show more selective signaling for environmental tolerance and stress signaling. We speculated that this result may be related to human activities and changes in environmental complexity. In conclusion, these results deepen our understanding of genetic diversity and selection signals in fishes, especially nearshore demersal fishes, and provide a theoretical and data basis for exploring potential mechanisms of local environmental adaptations in marine fishes.

## Methods

### Sample collection, DNA extraction, and sequencing

**Fig. 13:**
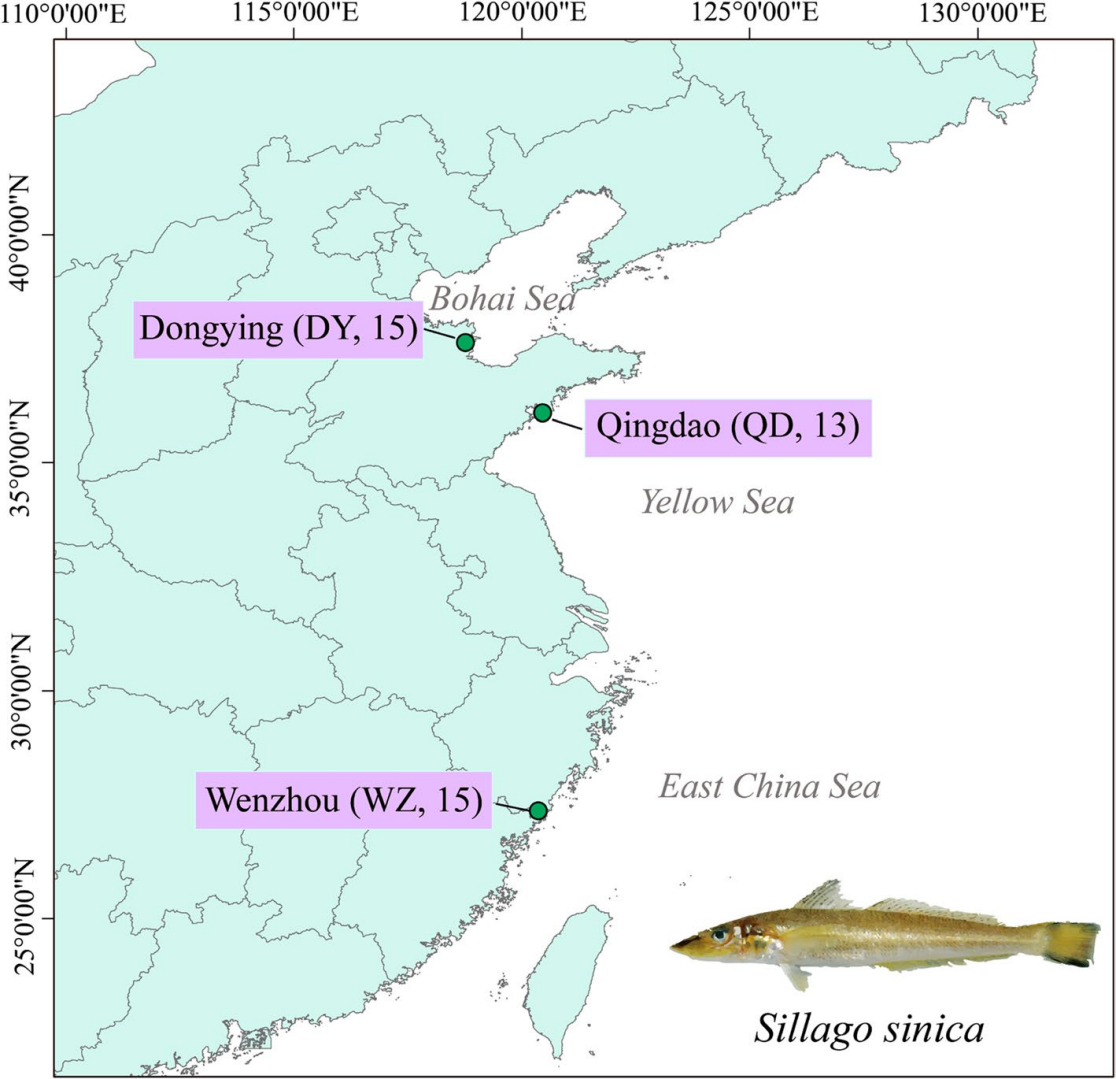
Distribution and population statistics of *S. sinica* sampling sites in this study

We collected 43 individuals of *S. sinica* from three populations distributed in the Yellow Sea, Bohai Sea and East China Sea (Fig. 13). A piece of muscle tissue was removed from each individual and stored in 95% ethanol or frozen for DNA extraction. Genomic DNA was extracted using the standard phenol–chloroform extraction procedure. For whole genome sequencing, at least 0.5 μg of genomic DNA was used to construct a library with an insert size of ~ 350 bp. Paired-end sequencing libraries (PE150) were constructed and sequenced on an Illumina HiSeq X Ten Sequencer (San Diego, CA, USA) by Novogene Bioinformatics Technology Co., Ltd., China.

### Sequencing data quality control and reads mapping

The raw image data obtained by sequencing were converted into raw sequence data (raw reads) by base calling, and raw reads contained linker sequences, low-quality bases and undetected bases (represented by N). We filtered this interference information and finally obtained clean reads. Xu et al. [22] have published the first complete reference genome of the *S. sinica* in 2018. The 534 Mb *S. sinica* genome assembly consists of 802 contigs with a contig N50 length of 2.6 Mb. Paired-end clean reads from each individual were mapped to the reference genome (http://gigadb.org/dataset/100490) using BWA software with the parameter “mem -t 4 -k 32 -M”, and a bam file was generated [47]. The BAM files were sorted using SAMtools and duplicate reads were removed [48].

### SNP calling, filtering and annotation

We called and filtered the raw SNPs according to GATK Best Practices from the alignment files [49]. SNPs were filtered based on the quality score of ≥ 20, QD (variant confidence/quality by depth) < 2.0, MQ (RMS mapping quality) < 40.0, FS (Phred-scaled *P* value using Fisher’s exact test to detect strand bias) > 60.0, SOR (symmetric odds ratio of 2 × 2 contingency table to detect strand bias) > 4.0, MQRankSum (Z score from Wilcoxon rank sum test of Alt vs. Ref read mapping qualities) < 12.5, or ReadPosRankSum (Z score from Wilcoxon rank sum test of Alt vs. Ref read position bias) < 8.0. ANNOVAR was used to annotate the genomic distribution of variants [50]. SNP variants were categorized based on their positions on the chromosome (including intergenic, exonic, intronic, splicing and 1-kb upstream and downstream regions) and on their effects (including stop codon gain or loss, synonymous and nonsynonymous).

### Population genetics analysis

After filtering, we generated a set of high-quality SNPs for the subsequent population genetic analysis. First, Treebest software (v.1.9.2) was used to calculate the distance matrix and construct the phylogenetic tree by the neighbor-joining (NJ) method. The number of bootstrap replicates was set to 1000 to test the reliability of the NJ clustering tree. PCA of whole-genome SNPs for all 43 individuals was performed with the PLINK software [51]. Population genetic structure and lineage information were assessed using PLINK and ADMIXTURE software [51, 52]. The number of assumed genetic clusters K ranged from 2 to 6. Linkage disequilibrium (LD) was calculated on SNP pairs within a 500-kb window using PopLDdecay software [53]. Linkage disequilibrium decay measured the distance at which Pearson’s correlation coefficient (R^2^) dropped to half of the maximum.

### Effective population size analysis of evolutionary history

The effective population size of the three populations was analysed using the PSMC model in the PSMC package [54]. The “fq2psmcfa” and “splitfa” tools in the PSMC software were used to create the input file for the PSMC modelling. The PSMC analysis command included the options “-N25” for the number of cycles of the algorithm, “-t15” as the upper limit for the most recent common ancestor (TMRCA), “-r5” for the initial θ/ρ, and “-p 4 + 25 * 2 + 4 + 6” for atomic intervals. The reconstructed population history was plotted using the “psmc_plot.pl” script. According to the method in the study by Han et al. [21]. The point mutation rate and the generation time were assumed to be 2.5 × 10^−8^ and 1 year respectively. TreeMix (v.1.13), which inferred the maximum-likelihood (ML) tree for the QD, DY and WZ populations. A window size of 1000 was used to account for linkage disequilibrium (–k) and “–global” to generate the ML tree. Migration events (–m 0 1 2) were sequentially added to the tree [55].

### Detection of selective sweeps

To uncover the genetic variants of warm temperate and subtropical *S. sinica* populations, we calculated the genome-wide distribution of population nucleotide diversity (π) and genetic differentiation indices (*F*st) using VCFtools with a window size of 40 kb and a step size of 20 kb [56]. The subtropical population (WZ) was set as the control group and the warm temperate populations (QD and DY) were set as the experimental group. The windows with the top 5% of values for the *F*st and π ratios were simultaneously used as a candidate region under strong selective sweeps. Based on these candidate regions, we localized to the corresponding SNPs and genes. We also performed the XP-EHH test for every SNP using the default settings of selscan v1.3.0 [57], which was designed to detect ongoing or nearly fixed selective sweeps by comparing haplotypes from 3 populations. For the XP-EHH selection scan, we extracted the genes involved in the 10 kb region above and below the selected loci. Genes with strong selection signals for each population were annotated using the eggNOG-mapper software [58]. Gene Ontology (GO) term classification and Kyoto Encyclopedia of Genes and Genomes (KEGG) pathway enrichment analysis were performed using OmicShare tools (http://www.omicshare.com/tools) [59–61].

## Data Availability

The sequence data have been deposited in the Short Read Archive (SRA) database of the National Center for Biotechnology Information (NCBI) under accession number PRJNA936440 (https://dataview.ncbi.nlm.nih.gov/object/PRJNA936440?reviewer=5jb7far1vjht86rjtbv86pgn02).

## Abbreviations

GWAS: Whole-genome resequencing and phenotype-specific genome-wide association study
NJ: Neighbor joining
PCA: Principal component analysis
PSMC: Pairwise sequentially markovian coalescent
LD: Linkage disequilibrium
MAF: Minimum allele frequency
TMRCA: The most recent common ancestor
GO: Gene Ontology
KEGG: Kyoto Encyclopedia of Genes and Genomes
SNPs: Single nucleotide polymorphisms
XP-EHH: Cross population extended haplotype homozogysity
